# Exploring computed tomography in ichnological analysis of cores from modern marine sediments

**DOI:** 10.1038/s41598-019-57028-z

**Published:** 2020-01-13

**Authors:** Javier Dorador, Francisco J. Rodríguez-Tovar, Jürgen Titschack

**Affiliations:** 1 Department of Earth Sciences, Royal Holloway University of London, Egham, Surrey TW20 0EX UK; 2 Departamento de Estratigrafía y Paleontología, Universidad de Granada, 18002 Granada, Spain; 3 MARUM – Centre for Marine Environmental Sciences, Bremen, Germany; 4 Marine Research Department, Senckenberg am Meer, 26382 Wilhelmshaven, Germany

**Keywords:** Palaeoceanography, Palaeontology, Sedimentology

## Abstract

Ichnological analysis is considered a very useful tool in several disciplines of Earth Sciences, including palaeoenvironmental studies and hydrocarbon exploration. Sediment cores provide excellent records, despite difficulties encountered during study runs due to specific core features. Previous studies using 2D images have proven the benefits of high-resolution image treatment in improving the visibility of ichnological features, but with limitations. 3D computed tomography (CT) techniques were applied to palaeoichnological studies in lithified cores and other disciplines of palaeontology to solve these limitations, but not used for ichnological studies in unconsolidated sediments due to the low density contrast between host sediment and trace fossils. In this study, a CT processing technique, previously tested in coral research, is applied to facilitate the characterisation of the ichnological signature of cores from modern marine soft sediments. This technique allows for the first time the isolation of burrows within these kinds of sediments and the differentiation of intervals based on burrow orientation. Data obtained from the technique are complemented with the ichnological information from conventional core description, thus providing a more complete characterisation of the trace fossil assemblage with additional ichnological properties such as burrow orientation and branching. This will improve palaeoenvironmental interpretations related to changes in energy or oxygenation, and the analysis of reservoir quality given the impact of burrows on porosity and permeability. Therefore, adopting CT to complement visual core description in the ichnological analysis of soft modern marine cores is a very informative approach.

## Introduction

Ichnological research is widely recognised as a very useful tool in the study of drilled marine cores in a wide range of fields. Biogenic structures, reflecting the behaviour of trace-makers in response to palaeoenvironmental conditions, provide valuable information into areas such as palaeoecology, facies analysis, palaeoenvironmental reconstructions, sequence stratigraphy and palaeoceanography^1–8^. In recent decades, the study of trace fossils has also become an essential part of reservoir and aquifer characterisation^9^. Numerous studies have proven that bioturbation is an important factor in the study of reservoir properties, affecting parameters such as porosity and permeability^10–18^. The key factors involved would be grain size re-distribution, morphology of trace fossils (e.g., shape, size, orientation, branching), burrow lining, infilling material of trace fossils, and bioturbation intensity^10,19–21^. For all these reasons, ichnology is seen as a powerful tool in Earth Science research and hydrocarbon exploration^4,13^.

Initially, ichnological studies were mostly based on outcrops and modern environments^21,22^, but the study of cores has gradually increased as the amount of core material recovered from oceanic expeditions became more abundant^9,23^. Core material makes it possible to analyse a continuous record, rarely observed in outcrops; yet it also entails some restrictions due to core features such as a narrow exposed surface or the exclusive observation of cross sections^24–27^.

Several techniques based on high-resolution digital image treatment have been applied in recent years, providing excellent results by improving the characterisation of particular ichnological features, such as the amount of bioturbation or the estimation of the penetration depth^9,26,28^. However, the determination of other ichnological properties in cores is still limited when they are based exclusively on 2D observations (e.g., three-dimensional shape, spatial distribution). Penetrative techniques—X-ray, Magnetic Resonance Imaging, or Computed Tomography (CT)—can be used to overcome such limitations^29–31^. These techniques have been successfully applied in lithified deposits or sediments with high density ferrugenised burrows^30,32,33^, as ferrugenised structures show high density contrast with respect to the host sediment, and thus can be easily isolated.

Still, the use of these techniques is quite rare in the ichnological analysis of soft sediments cores, where the density contrast is usually low. One of the first ichnological analyses which used CT on cores from unconsolidated deep-sea sediments was published in the 90’s and it provided information on trace fossil assemblages and the 3D distribution of biogenic structures^34^. In spite of their good results, isolation of trace fossils was not conducted, probably due to technical limitations and the low density contrast between trace fossils and the surrounding sediment. Similar results obtained in cores where density contrast is low (e.g., mudstone) show CT techniques to be less effective^35^.

After several years working on high-resolution image treatment as a useful tool applied on ichnological research^27^, here we present a next step in the ichnological analysis of cores from modern marine soft sediments based on the application of CT. This CT processing technique was previously applied on coral research from similar type of soft, unlithified, sediments^36^. The main goal is to explore the potential/capability of the technique in obtaining ichnological data (i.e., trace fossil assemblage, amount of bioturbation and other ichnological features) and the isolation of 3D structures. Data obtained from the CT processing technique are then combined with those acquired from treated high-resolution images, as an integrative approach for ichnological analysis.

**Figure 1:**
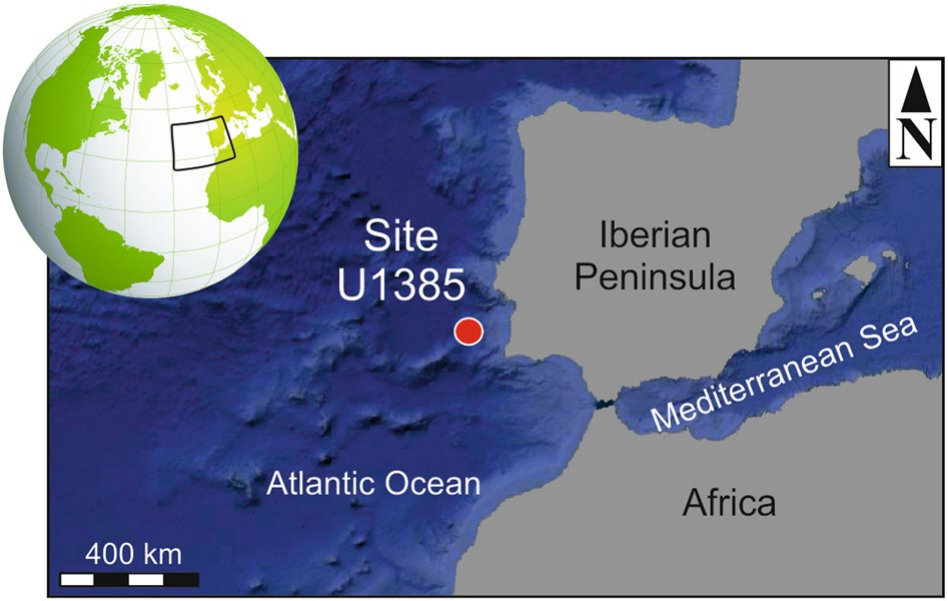
Location of Site U1385. Bathymetry map data: Google, Landsat/Copernicus. Globe image by Pixabay.

This study was conducted on six core sections (~1.50 m long each) from hemipelagic sediments drilled at Site U1385, located in the Western Iberian Margin (Fig. 1), from IODP Expedition 339. These cores are composed on Pleistocene to Holocene highly bioturbated muds and clays without sedimentary structures^37^. They were selected due to the presence of well-defined ichnofabrics, characterised in previous ichnological studies based on the application of a digital image treatment to high-resolution scanned images^25,27^.

## Method Background

**Figure 2:**
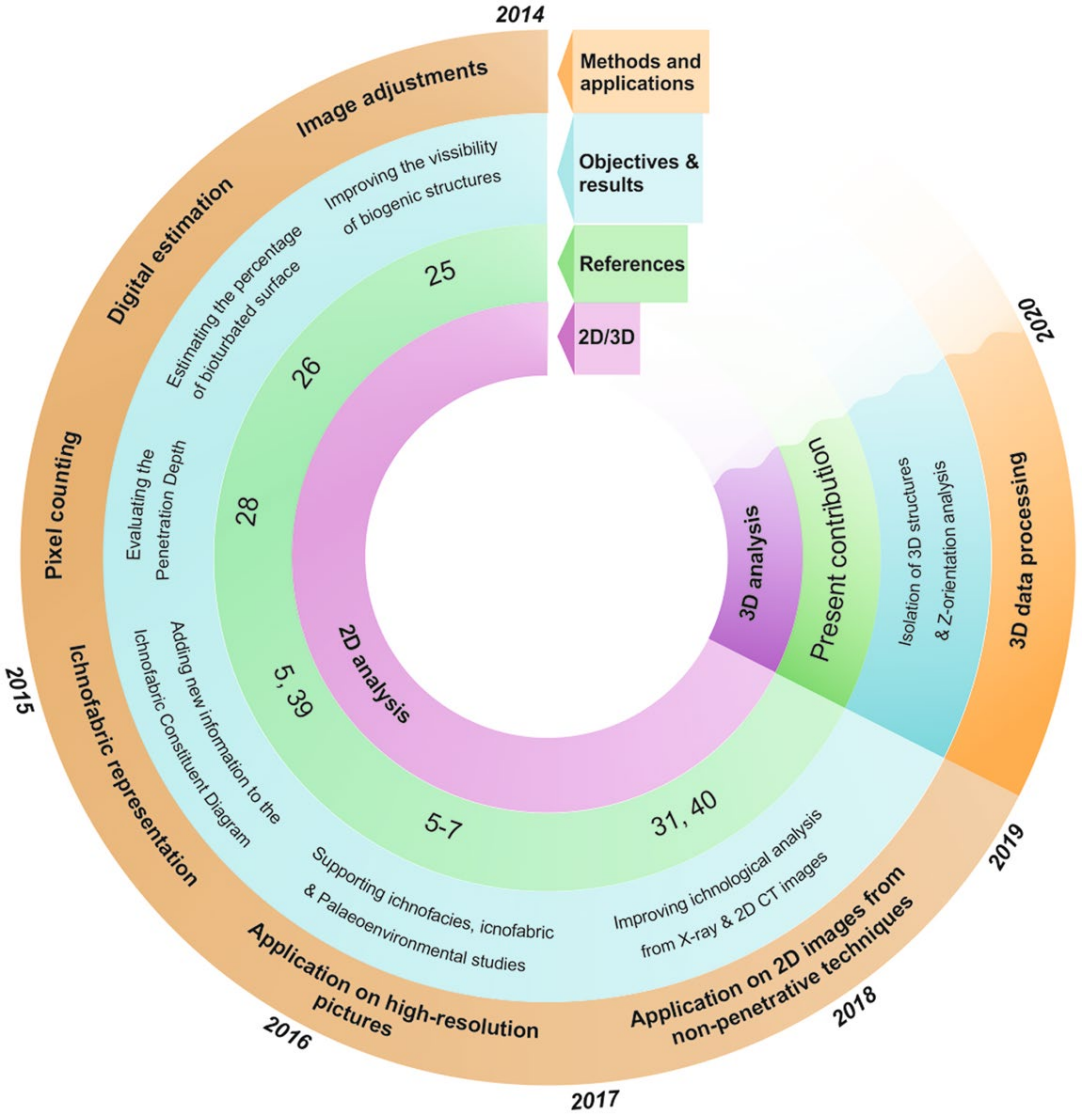
Schematic diagram of the method background and the present contribution.

High-resolution image treatment has already been proven as a powerful tool for ichnological analysis of cores from unconsolidated sediments^25,26,28^. Several techniques were developed during the last decade to help improve trace fossil visualisation by some image adjustments^25^, support the quantification of bioturbation by the application of several software methods^26^, and estimate the penetration depth after a quantitative pixel analysis^28^ (Fig. 2). They have been successfully applied for ichnofacies characterisation^38^, ichnofabric approach^5,39^, and palaeoenvironmental studies^5–7^. These methods are effective for high-resolution pictures, but have also provided good results on 2D images obtained from non-penetrative techniques (i.e., X-ray or CT)^31,40^. All these advances have benefited ichnological studies on unconsolidated sediments, solving the limitations due to poor visibility of traces fossils in this kind of sediments, however it is just limited to a 2D visualization of the exposed surface. This limitation was solved using 3D CT techniques when working with lithified cores, allowing the isolation of 3D structures, but this process is not possible for unconsolidated sediments due to the low density contrast. Here, for the first time, we were able to utilise a CT data treatment processing technique, explained in the *Methods* section, to isolate biogenic structures in marine soft sediments and to obtain extra ichnological information and their dominant orientation, which is useful for sedimentary basin analysis, palaeoenvironmental and reservoir quality applications.

## Results

**Figure 3:**
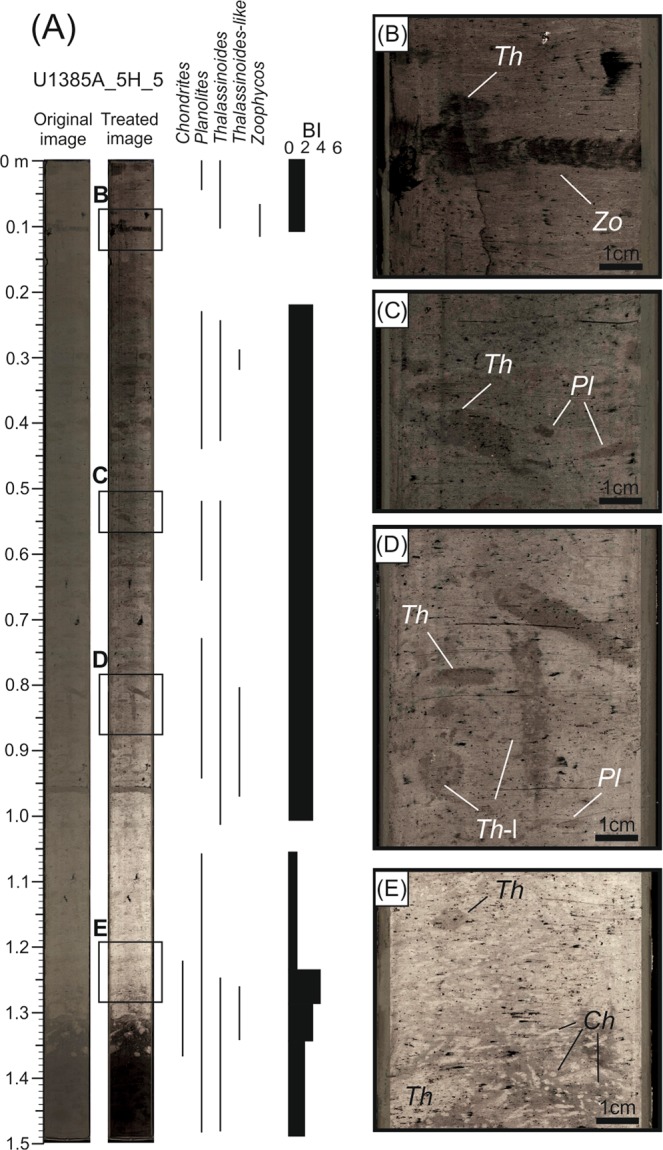
Ichnological content and Bioturbation Index (BI) based on high-resolution treated image from core section U1385A_5H_5. *Ch, Chondrites; Pl, Planolites; Th, Thalassinoides; Th-l; Thalassinoides-like; Zo, Zoophycos*.

**Figure 4:**
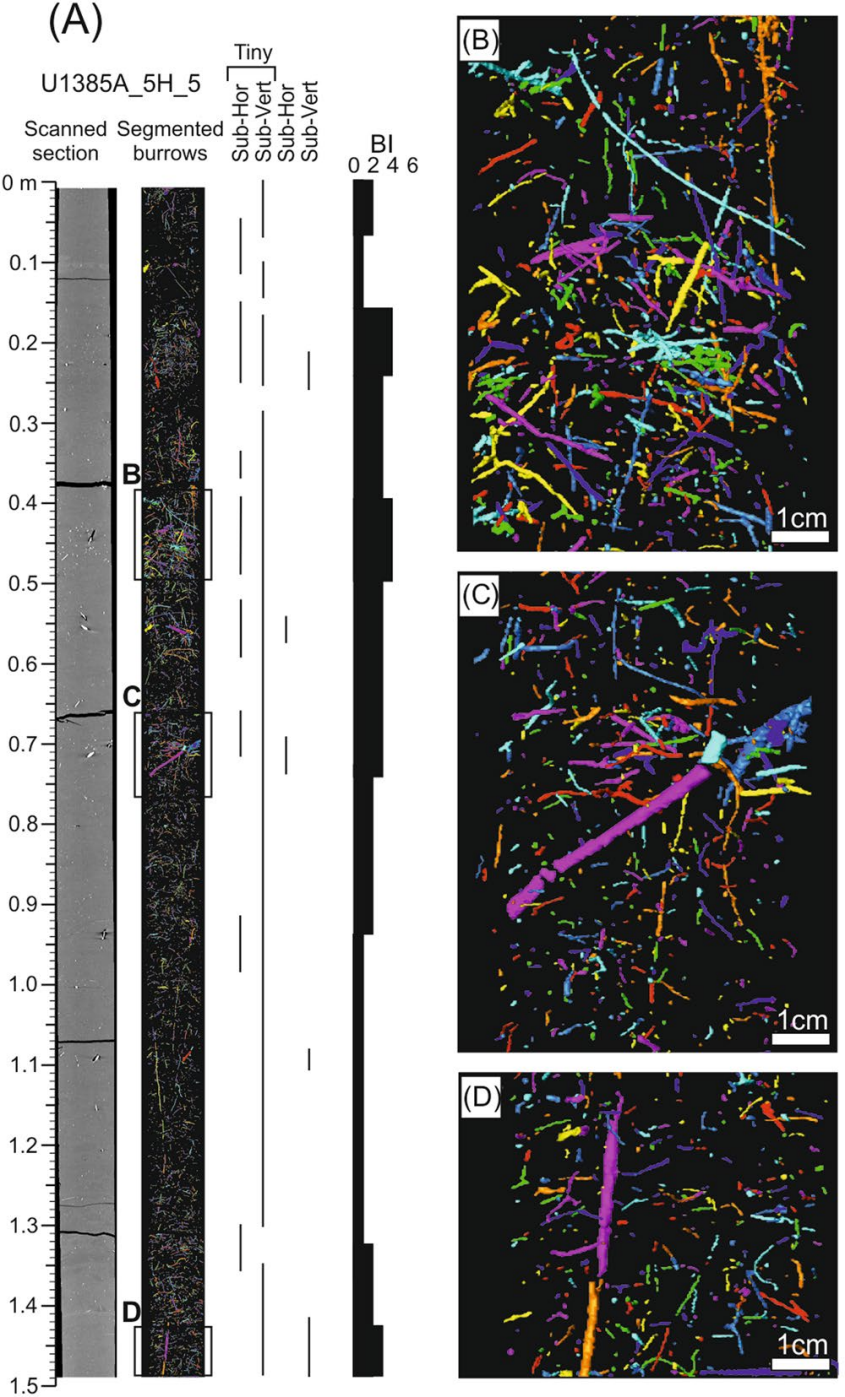
Ichnological content and Bioturbation Index (BI) based on segmented burrows by CT processing from core section U1385A_5H_5.

**Figure 5:**
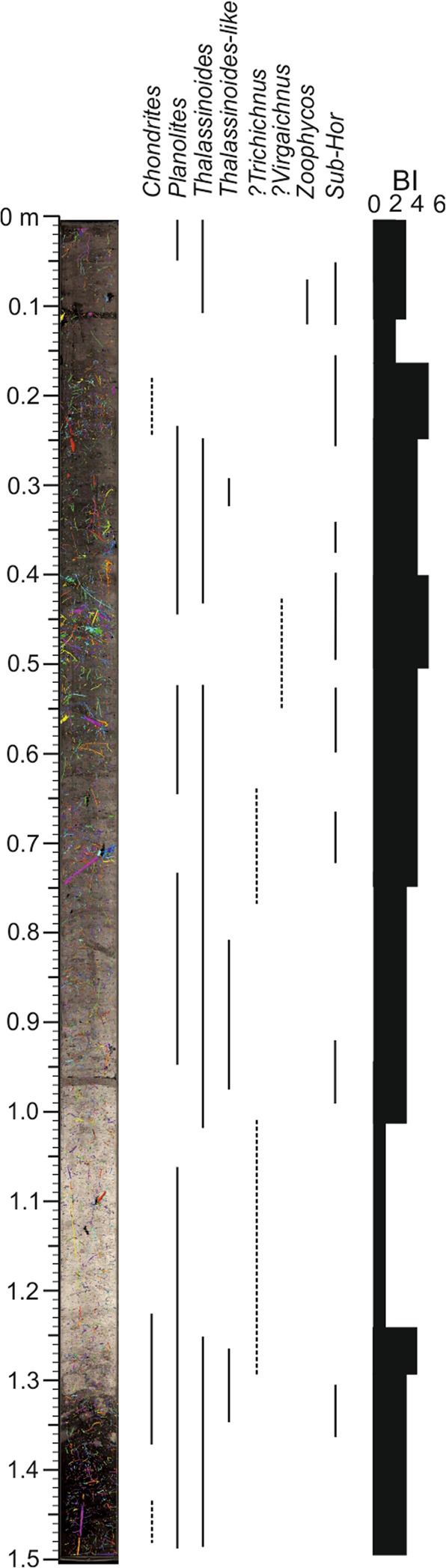
Ichnological content and Bioturbation Index (BI) from core section U1385A_5H_5 combining high-resolution treated images and segmented burrows.

**Figure 6:**
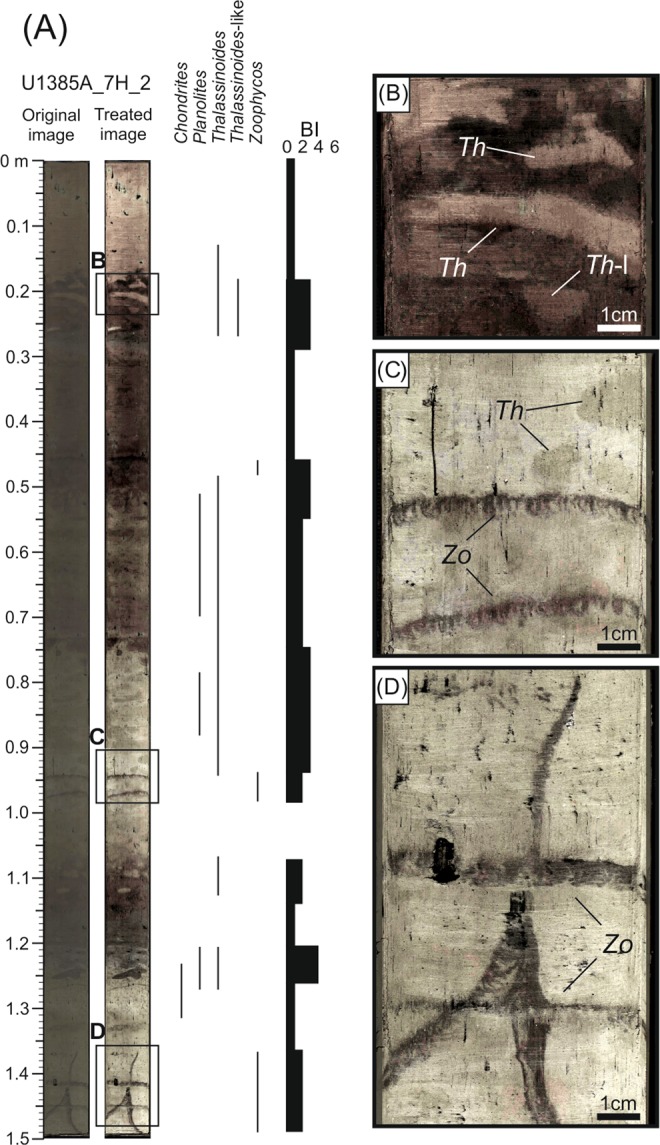
Ichnological content and Bioturbation Index (BI) based on high-resolution treated image from core section U1385A_7H_2. *Ch, Chondrites; Pl, Planolites; Th, Thalassinoides; Th-l; Thalassinoides-like; Zo, Zoophycos*.

**Figure 7:**
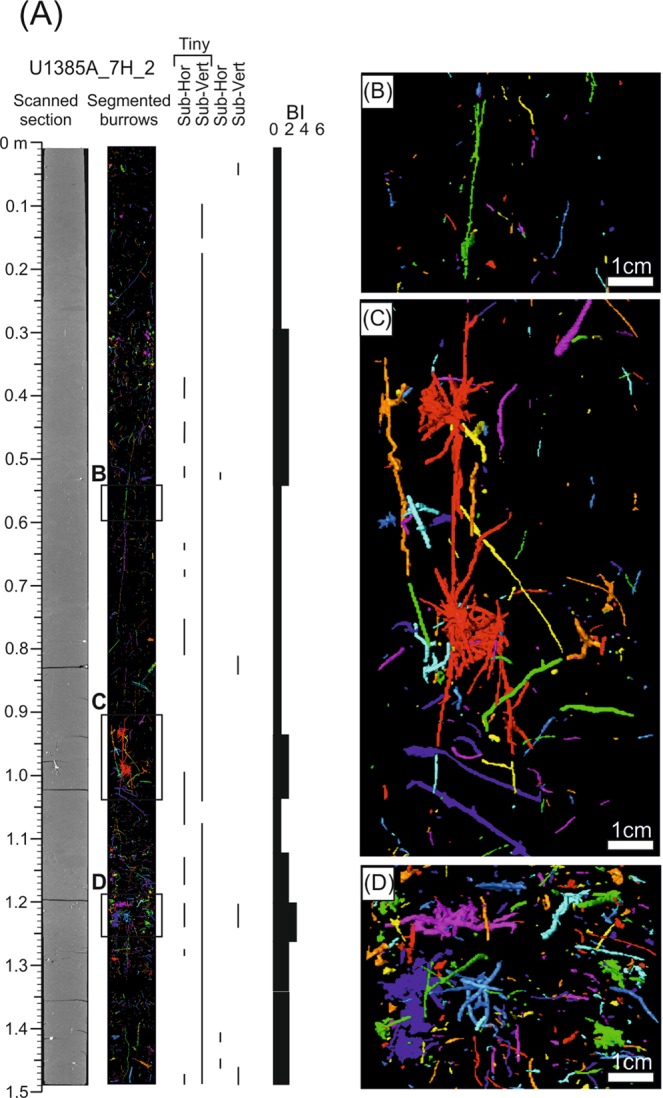
Ichnological content and Bioturbation Index (BI) based on segmented burrows by CT processing from core section U1385A_7H_2.

**Figure 8:**
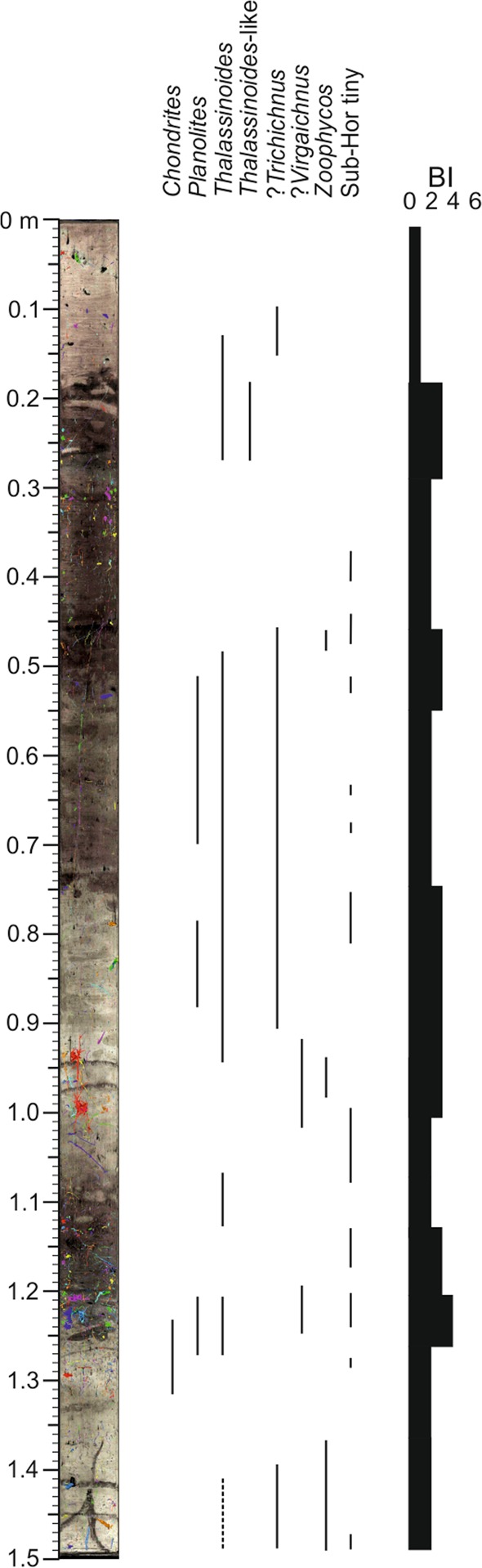
Ichnological content and Bioturbation Index (BI) from core section U1385A_7H_2 combining high resolution treated images and segmented burrows.

**Figure 9:**
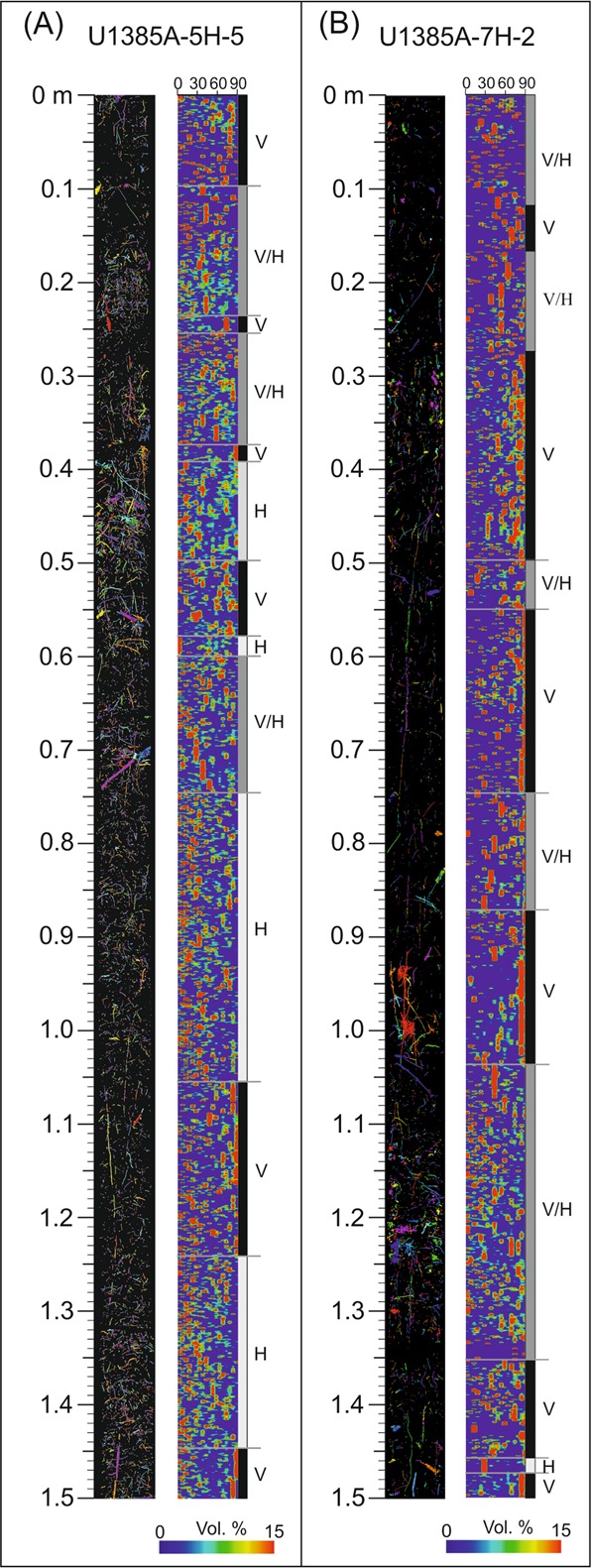
Isolated burrows and z-orientation analysis with interval characterisation from core section U1385A_5H_5 (**A**) and U1385A_7H_2 (**B**) H, intervals where horizontal burrows are dominant; V, intervals where vertical burrows are dominant; V/H, intervals where there is no dominant orientation.

An initial analysis of the scanned core sections reveals that not all trace fossils could be segmented by CT data because of the low density contrast between the infilling material of trace fossils and the surrounding sediment. Thus, we have selected two illustrative core sections, U_1385A_5H_5 and U_1385A_7H_2, to analyse the trace fossil assemblage, degree of bioturbation, and relationship between vertical and horizontal burrows (Figs. 3–9, Table 1).

**Table 1:** Trace fossils assemblages and Bioturbation Index (BI) obtained from high resolution images, CT isolated burrows and combining both methods for the two studied sections.

	High-resolution images		CT isolated burrows		Combined results	
	Trace fossils assemblage	BI	Trace fossils assemblage	BI	Trace fossils assemblage	BI
U_1385A_5H_5	*Chondrites*, *Planolites*, *Thalassinoides*, *Thalassinoides*-like, *Zoophycos*	0–4	*Trichichnus*, *Virgaichnus*	1–4	*Chondrites*, *Planolites*, *Thalassinoides*, *Thalassinoides*-like, *Trichichnuss*, *Virgaichnus*, *Zoophycos*	1–5
U_1385A_7H_2	*Chondrites*, *Planolites*, *Thalassinoides*, *Thalassinoides*-like, *Zoophycos*	1–4	*Trichichnus*, *Virgaichnus*	1–3	*Chondrites*, *Planolites*, *Thalassinoides*, *Thalassinoides*-like, *Trichichnuss*, *Virgaichnus*, *Zoophycos*	1–4

### Core section U_1385A_5H_5

The first case is from the 1.5-meter-long U_1385A_5H_5 core section. Five ichnotaxa were recognised at the ichnogenus level (*Chondrites*, *Planolites*, *Thalassinoides*, *Thalassinoides*-like and *Zoophycos*) from the high-resolution treated image (Fig. 3 and Table 1). Burrows isolation did not allow this assemblage to be discerned in CT images, but sub-horizontal and sub-vertical burrows can be clearly visualised and grouped according to their diameters, classifying as tiny burrows those with a diameter under 0.5 cm (Fig. 4). Regarding the Bioturbation Index (BI) in the digital treated image (Fig. 3 and Table 1), the bottom part shows an increase from low (BI = 2) to medium (BI = 4), and then a decrease upward (BI = 1). From that point to the top, the core shows a medium Bioturbation Index (BI = 3), which is lower in the upper part (BI = 2), with a short interval between them having no bioturbation (BI = 0). The BI obtained from CT images shows a similar general pattern, but with some notable differences in particular intervals, such as in the upper part of the section where it is characterised by a BI = 0 (absence of trace fossils) from the digital images, but showing a medium degree of bioturbation (BI = 4) according to CT images. In contrast, the lower part of the section, which is highly bioturbated in the digital treated image (Fig. 3), shows a low BI in the CT image (Fig. 4).

Combining information from the two methods provides an enhanced characterisation of some ichnotaxa and a more precise BI determination. Concretely, some vertical tiny burrows differentiated with CT can be associated to ?*Trichichnus*, while complex structures of tiny burrows can be linked to ?*Virgaichnus* (Fig. 5) as observed in core analysis^9^. *Trichichnus* is described as cylindrical tiny (0.1–1.0 mm diameter) vertical burrows that can reach several meters in length (often more than 20 cm) and is commonly observed in hemipelagic deposits. *Virgaichnus* is defined as a complex tiny burrow (less than 1 mm diameter) system, showing a pinch and swell morphology, that is typical of conditions with low sedimentation rate^9,41^. Some of the isolated tiny burrows could be associated to ‘*Mycellia*’, being several centimeters long and randomly oriented^30,41–44^, yet we chose to be conservative and decided not to pursue a very detailed ichnotaxonomical classification based on CT.

The values obtained for BI were modified after study of the CT sections, considering the structures identified by both methods. The degree of bioturbation is generally medium (BI = 3–4), except for a low bioturbated interval in the bottom part (BI = 1) and some short intervals from the upper half where bioturbation reaches high values (BI = 5) (Fig. 5).

### Core section U_1385A_7H_2

The second case corresponds to core section U_1385A_7H_2. Analysis of high-resolution digital images reveals a trace fossils assemblage similar to U_1385A_5H_5, consisting of *Chondrites*, *Planolites*, *Thalassinoides*, *Thalassinoides*-like and *Zoophycos* (Fig. 6 and Table 1). Regarding isolated burrows after CT processing, sub-vertical burrows are dominant throughout the section, especially the tiny ones, while sub-horizontal structures play a secondary role (Fig. 7). Despite limitations in the characterisation based on isolated trace fossils, some of the tiny sub-vertical burrows can be assigned to ?*Trichichnus* and the complex 3-D tiny structures to ?*Virgaichnus* (Fig. 8 and Table 1). Sub-horizontal tiny burrows are clearly differentiated, though an ichnotaxonomical classification is not possible; yet larger (diameter >0.5 cm) isolated sub-horizontal burrows can be assigned tentatively to *Thalassinoides*.

The degree of bioturbation obtained from digital images is generally low (BI = 1–2) excluding some intervals where there is an increase to medium (BI = 3–4), especially around 125 cm depth (Fig. 6). These data are consistent with observations of the degree of bioturbation from CT images, where BI = 1–2 are dominant throughout the core section (Fig. 7).

After recalculating BI values in view of structures identified by both methods, the degree of bioturbation in general was seen to be low/medium (BI = 2–3), excluding a short interval where it is high (BI = 4) and the upper part of the section where it is low (Fig. 8).

### Z-orientation

Analysis of the z-orientation of isolated burrows derived from CT processing allows for a qualitative differentiation of intervals. It is thus possible to characterise the intervals dominated by vertical or horizontal burrows, or mixed intervals lacking a clearly dominant orientation (Fig. 9). In the studied U_1385A_5H_5 core section, intervals of dominant horizontal (H in Fig. 9A) or vertical (V in Fig. 9A) burrows are clearly distinguished, although these show a similar abundance of horizontal and vertical structures and a variable distribution without any trend throughout the section.

In the core section U_1385A_7H_2, the analysis of the z-orientation shows a clear vertical burrow orientation, with dominance of vertical burrows or mixed intervals (Fig. 9B). Moreover, it is remarkable that some of the segmented vertical burrows are over 40 centimetres long, showing a high penetration depth.

## Discussion

The application of CT techniques on cores from modern marine sediments, or soft cores, has provided useful information on trace fossil assemblage, amount of bioturbation and orientation of burrows, especially for cases with high density contrast between the infilling material of trace fossils and the surrounding sediment. This information can be of interest in terms of ichnotaxonomical characterisation, and inferred interpretations.

### Ichnotaxonomical characterisation

Significant differences in trace fossil identification were found by comparing results obtained from treated digital images and CT data. On one hand, most trace fossils that were clearly identified in high-resolution digital images were not recognised by means of CT processing. As example, *Zoophycos* was clearly identified several times along the studied sections (see a clear example in the bottom part of U1385A_7H_2 section), but this ichnotaxon was not observed in CT segmented structures (Figs. 6 and 7). On the other hand, burrows seen to be segmented using CT were not observed in the exposed core surface in the high-resolution images. A clear example can be observed in section U1385A_7H_2 where 3D complex structures are identified in the middle part of the section, but they are not observed in the high-resolution images (Figs. 6 and 7).

A higher diversity of the trace fossil assemblage discerned in the treated digital images with respect to the CT information could be attributed to three major factors. The density difference between the infilling material of burrows and host sediment, characterised as calcareous muds and calcareous clays^37^, may be too low. Commonly, penetrative techniques such as X-ray or CT allow only the visualisation of burrows with high density contrast (e.g., pyritised burrows, empty burrows) which most often do not represent the complete trace fossil assemblage^30^. Secondly, the resolution of CT scan (0.351 × 0.351 × 0.3 mm in this case) is commonly too coarse to segment very tiny burrows and to identify some ichnological features (e.g., spreiten or backfills). Furthermore, the ichnotaxonomical identification of trace fossils based on CT data is not easily achieved, while 3D overall shape, size, and orientation can be observed, other ichnotaxobases such as lining, infilling material, or internal structure cannot be distinguished^3,24,45–47^.

The presence of segmented burrows using CT, which are not registered in the treated high-resolution digital images, can be a consequence of slabbed core features (i.e., just one longitudinal slide is observed). The exposed surface seen in the digital images is a vertical slide, which facilitates the observation of mostly (sub-)horizontal structures, whereas vertical forms are likely to go unobserved^23^. Therefore, it is more common to find cross sections of trace fossils with (sub-) horizontal shape or dominant (sub-) horizontal arrangement, as in the case of the horizontal tubes of *Thalassinoides*^48^ or the *Zoophycos* helicoidal structure^49^. Meanwhile, it is comparatively rare to observe a cross section of dominantly vertical ichnotaxa, such as *Skolithos* or *Trichichnus*^9^.

Accordingly, the combination of both techniques —high-resolution images and CT processing for segmented burrows—leads to a better overall characterisation of the trace fossil assemblage. In the studied cases, the trace fossil assemblage is composed of *Chondrites*, *Planolites*, *Thalassinoides*, *Thalassinoides*-like and *Zoophycos* according to image descriptions, and could be complemented by ?*Trichichnus*, ?*Virgaichnus* and other unidentified tiny burrows characterised by CT.

### Palaeoenvironmental analysis

Site U1385 is located off the west Iberian margin on a spur, the Promontorio dos Principes de Avis, along the continental slope of the southwestern Iberian margin^37^ (Fig. 1). The section drilled at Site U1385 correspond to a typical hemipelagic continental margin succession deposited under normal marine conditions, with good bottom and pore-water oxygenation, and organic matter availability^5,37^. Ichnological features including trace fossil assemblage, amount of bioturbation and dominant orientation can be used to approach environmental parameters, such as oxygenation, nutrient availability, sedimentation rate, and hydrodynamic energy^2–5^.

In the case study, the identification of ?*Trichichnus* was possible only with CT processing (Figs. 4 and 7). The presence of *Trichichnus* is commonly associated with low oxygen sediments and bioelectric bacterial activity^9,32,50,51^. Moreover, high density contrast between burrows and host sediment has sometimes been associated to the presence of pyrite in burrows^32,39^. Burrow pyritisation may be related with an organic active filling generated by trace-makers associated with low oxygen waters and organic-rich sediments, which is also consistent with the ethological interpretation of *Trichichnus*^9^. This interpretation of low oxygen conditions within sediment and organic-rich sediments, at least in some intervals of the studied sections, enhances previous palaeoenvironmental interpretations based exclusively on the trace fossil assemblage differentiated in high-resolution digital images^5^.

As for Bioturbation Index, some differences became apparent when comparing data obtained from either technique with those from a combined analysis. The degree of bioturbation obtained by each single technique is lower than that derived from the combination. For instance, in a short interval from the upper part of the core section U1385A_5H_5 there is no bioturbation (BI = 0), but after combining data from both techniques, this interval shows a low to medium bioturbation index (BI = 2–4). Considering that the quantification of bioturbation may be linked to bottom conditions affecting the trace-maker community^4,9^, the lower estimation would give rise to misinterpretation of these palaeoenvironmental conditions. Thus, absence of bioturbation (BI = 0) could be related to an inhabitable environment for trace-maker community due to unfavorable conditions (e.g., anoxia, absence of nutrients, high energy, among others); this interpretation is discarded when a BI = 2–4 is considered.

Palaeoenvironmental interpretation can be further enhanced by z-orientation analysis. Orientation of trace fossils within sediment has been widely used to interpret palaeoenvironmental conditions, as it can be affected by parameters such as salinity, temperature or nutrients^52^ and it has been commonly used to interpret hydrodynamic energy conditions^53,54^. Settings characterised by high energy conditions are commonly dominated by vertical burrows, mostly associated with suspension-feeders, reflecting an escape strategy^47^. Low energy areas are largely dominated by horizontal structures produced by detritus- or deposit-feeders^55^. In the studied cores, differentiation of stratigraphic intervals based on dominant orientation could be used to infer minor variations in energy or nutrients within hemipelagic deposits. These changes are relatively frequent in the bottom half of the U1385A_5H_5 section, where there is a clear alternation between horizontal and vertical intervals, whereas the changes are minor in section U1385A_7H_2 where vertical burrows prevail over horizontal ones throughout the section.

## Conclusions

For the first time, the isolation of 3D biogenic structures in cores of unconsolidated sediments has been possible, allowing more information on ichnological features, such as the dominant z-orientation, to be obtained. The orientation of biogenic structures has been considered as a tool in sedimentary basin research as an index of depositional parameters, such as hydrodynamic conditions, proximal to distal gradients or sedimentation rate, among others. In regards to hydrocarbon exploration, the dominant orientation can also determine vertical and/or horizontal permeability, which controls fluid flow.

The combination of this new information obtained from the 3D CT data with information obtained from existing techniques such as the 2D high-resolution images could provide valuable information for the characterisation of palaeoenvironmental parameters in further studies. Moreover, integration of core images and CT data could potentially serve as a proxy to analyse the effect of bioturbation on petrophysical properties including porosity and permeability values, which play an important role in hydrocarbon exploration. However, further analyses are necessary to support the effectiveness of this technique in the industrial aspect.

Considering these applications, the relevance of CT for ichnological analysis of cores from modern marine sediments can be upheld as a promising powerful resource for future studies, particularly when integrated with other ichnological approaches.

## Methods

The ichnological assemblage and the amount of bioturbation are analysed in detail. The amount of bioturbation was characterised and referred throughout the manuscript in terms of the Bioturbation Index^56,57^, following Reineck’s scale from 0 (no bioturbation) to 6 (completely bioturbated), considering discrete trace fossils and overlooking a mottled background.

For comparison, computed tomography was applied on selected 1.5 m core sections.

**Figure 10:**
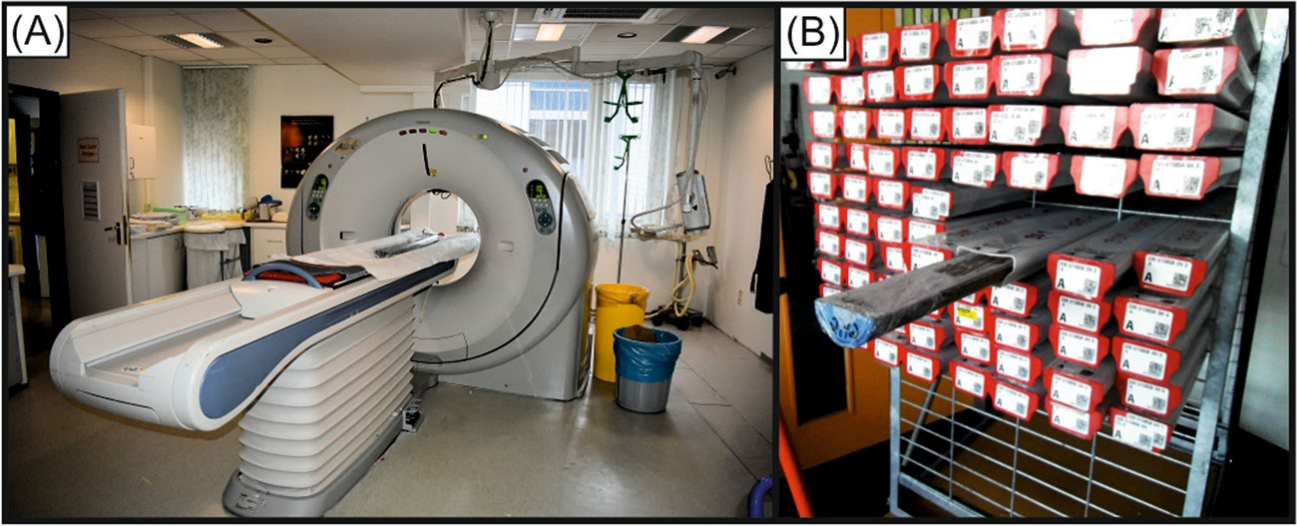
Computed tomography scan used in the present study (**A**) and some examples of the slabbed core sections from Site U1385 (**B**).

Archive halves (6.2 cm diameter) of the six selected core sections (U1385A-4H-5A, U1385A-5H-5A, U1385A-7H-2A, U1385A-8H-1A, U1385A-11H-2A, U1385B 12H-1A) were scanned by a Toshiba Aquilion 64 computer tomograph (Fig. 10) at the Klinikum Bremen-Mitte (Bremen, Germany) with an x-ray source voltage of 120 kV and a current of 600 mA. Images were reconstructed using Toshiba’s patented helical cone beam reconstruction technique (TCOT) and are provided in DICOM-format. The CT image stacks have a resolution of 0.35 mm in x- and y-direction and 0.5 mm resolution in z-direction (0.351 mm reconstruction unit).

The data were treated using a time-consuming and complex processing with the ZIB edition of Amira software (version 2017.02)^58^. Within Amira, the core liners (showing similar x-ray attenuation than the sediments and did not affect data quality of the CT scans), including about 2 mm of the core rims (to avoid drilling artefacts), were deleted from the dataset. Pyrite-filled burrows with diameters >~1 mm were segmented with a marker-based watershed algorithm (Watershed (Skeleton) module). Markers were set with the Segmentation Editor by threshold segmentation. The segmented burrows were separated from each other with the *ConnectedComponents* module. Afterwards, every trace fossil were parameterised with the *ShapeAnalysis* module. The determined z-orientation (horizontal = 0°; vertical = 90°) of the trace lengths were further used for a trace fossil orientation analysis. Therefore, the z-orientation of every trace within one CT-slice were considered and the obtained results were assigned to the respective slice position. The results (unit: volume-% of all segmented trace fossils) were exported into a spreadsheet. For the evaluation of the trace segmentation (i.e., isolation), the trace fossils were visualised with the *SurfaceGen* and *SurfaceView* module. Each identified trace was displayed with a different colour (for further details on the methodology see also^36,59^).

The combination of results from both techniques is carried out by generating a new image with the plotting of isolated burrows obtained by CT over the scanned core images.
